# Competition in the Periphytic Algal Community during the Colonization Process: Evidence from the World’s Largest Water Diversion Project

**DOI:** 10.3390/plants13152067

**Published:** 2024-07-26

**Authors:** Yuxuan Zhu, Xiaojie Tu, Yonghong Bi, Gaofei Song, Wujuan Mi

**Affiliations:** 1 State Key Laboratory of Freshwater Ecology and Biotechnology, Institute of Hydrobiology, Chinese Academy of Sciences, Wuhan 430072, China; yxzhu@ihb.ac.cn (Y.Z.); biyh@ihb.ac.cn (Y.B.); song@ihb.ac.cn (G.S.); 2 Hubei Key Laboratory of Resources and Eco-Environment Geology (Hubei Geological Bureau), Wuhan 430034, China; txjshining525@163.com

**Keywords:** periphytic algae, colonization process, niche, interspecific competition, South to North Water Diversion Project

## Abstract

Periphytic algal colonization is common in aquatic systems, but its interspecific competition remains poorly understood. In order to fill the gap, the process of periphytic algal colonization in the Middle Route of the South to North Water Diversion Project was studied. The results showed that the process was divided into three stages: the initial colonization stage (T1, 3–6 days), community formation stage (T2, 12–18 days) and primary succession stage (T3, 24–27 days). In T1, the dominant species were *Diatoma vulgaris* (Bory), *Navicula phyllepta* (Kützing) and *Fragilaria amphicephaloides* (Lange-Bertalot) belonging to Heterokontophyta; these species boasted wide niche widths (NWs), low niche overlap (NO) and low ecological response rates (ERRs). In T2, the dominant species were *Diatoma vulgaris*, *Cymbella affinis* (Kützing), *Navicula phyllepta*, *Fragilaria amphicephaloides*, *Gogorevia exilis* (Kützing), *Melosira varians* (C.Agardh), *Phormidium willei* (N.L.Gardner) and *Cladophora rivularis* (Kuntze). These species displayed wider NWs, lower NO, and lower ERRs than those in T1. In T3, the dominant species were *Diatoma vulgaris*, *Cymbella affinis*, *Navicula phyllepta*, *Fragilaria amphicephaloides*, *Achnanthes exigu* (Grunow), etc. Among them, Heterokontophyta such as *Diatoma vulgaris* and *Cymbella affinis* had a competitive advantage based on NWs and ERRs. Cyanobacteria like *Phormidium willei* lost their dominant status due to the narrower NW and the increased NO. It could be concluded the interspecific competition became fiercer and shaped the colonization process; this study will be helpful in understanding the colonization of periphytic algal communities.

## 1. Introduction

Periphytic algae constitute a diverse group of organisms that play pivotal ecological roles in aquatic ecosystems [1,2]. They encompass a broad range of species, inhabit diverse habitats and attach to substrates in various manners [3,4]. Periphytic algae possess numerous ecological functions, including primary production, chemical regulation, providing biological habitats and serving as environmental indicator species [5,6]. Furthermore, they can be viewed as solar biogeochemical reactors, hydraulic roughness factors, early warning systems for environmental degradation and repositories of biodiversity [7,8]. The rapid proliferation and senescence of periphytic algae significantly alter nutrient structures, water quality and interspecific relationships among aquatic organisms [9]. Additionally, they can block water delivery pipes, emit unpleasant odors and pose a threat to water supply safety; the harmful microorganisms associated with periphytic algae increase health risks for humans [3,10]. On the other hand, periphytic algae are influenced by a myriad of environmental factors, which shape the structure, function and appearance of periphytic algal communities [5,6]. Understanding the influencing factors, ecological processes and ecological functions of periphytic algae is crucial for their effective management.

The colonization process is a crucial ecological process for periphytic algae [7,8,11]. This process is influenced by several physical and chemical factors, such as water velocity, light intensity, nutrient availability and substrate type [12,13]. Moreover, bacteria, fungi and protozoa, which are closely associated with periphytic algae, can also affect the colonization process [14,15]. Despite the influence of physical, chemical and biological factors on colonization processes, there is still limited understanding of the mechanisms governing interspecific interactions [16,17]. Therefore, further research into the interspecific relationships between periphytic algae during colonization is needed.

The Middle Route of the South-to-North Water Diversion Project (MRP) is the largest water transfer project in the world, spanning a total length of 1432 km, and aims to provide clean drinking water for 200 million people [18,19]. The MRP comprises a lengthy canal and intricate hydraulic structures, transporting drinking water to Henan, Hebei, Beijing and Tianjin. Since its inception in 2014, the relatively short operating time and closed engineering system have reduced the complexity of the MRP ecosystem. However, the numerous hydraulic structures and engineered perturbations have resulted in significant variations in the periphytic algal community [20,21]. Currently, there are limited studies pertaining to periphytic algae in the MRP [22]. Consequently, gaining insight into the periphytic algae community in the MRP is imperative to maintain stable water quality and water transmission safety.

In this study, we aim to simultaneously investigate the community structure and environmental influencing factors of periphytic algae in the MRP. This is the first attempt to utilize niche theory to analyze the colonization of periphytic algal communities in the world’s largest water diversion canal. The objectives of this work are to (i) comprehend the colonization patterns of the periphytic algal community in the water diversion canal and (ii) examine how the niche of periphytic algae evolves during colonization. This study intends to contribute to our understanding of the development of periphytic algal communities in water conservancy projects.

## 2. Results

### 2.1. The Colonization Process

**Figure 1:**
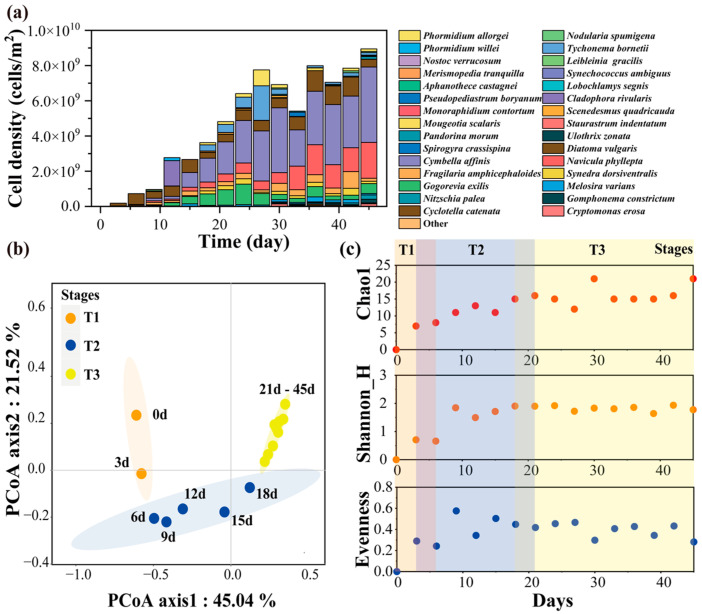
The community structure, colonization stages and α-diversity during the colonization of periphytic algal community ((**a**) community structure, (**b**) stages, (**c**) α-diversity).

A total of 150 algae species were observed, with Heterokontophyta (76–95%), Chlorophyta (2–7%), Cyanobacteria (7–11%), Charophyta (1–2%) and Cryptista (1%) representing the most common periphytic algal phyla. Among these, 30 species were identified as the most prevalent during the colonization process. Notably, Heterokontophyta emerged first, followed by Cyanobacteria and Chlorophyta. The number of algal species displayed a gradual increase, while the cell density initially rose and then declined after the 27th day (Figure 1a).

A Bray–Curtis-based Principal Coordinates Analysis (PCoA) revealed that the colonization process of the periphytic algal community could be delineated into three distinct stages: the initial colonization stage (T1, 3–6 days), the community formation stage (T2, 12–18 days) and the primary succession stage (T3, 24–27 days) (Figure 1a,b). The community displayed significant differences among different stages (PERMANOVA, *F* = 7.62, *p* = 0.001). The community structure dissimilarity (Xd) varied markedly among colonization stages, being significantly higher (PERMANOVA, *p* < 0.005) in T2 (Xd =0.79) than in T3 (Xd = 0.58). The dominant species in different stages were significantly different: *Diatoma vulgaris* (Bory), *Navicula phyllepta* (Kützing) and *Fragilaria amphicephaloides* (Lange-Bertalot) were the dominant species in T1; *Diatoma vulgaris*, *Cymbella affinis* (Kützing), *Navicula phyllepta*, *Fragilaria amphicephaloides*, *Gogorevia exilis* (Kützing), *Melosira varians* (C.Agardh), *Phormidium willei* (N.L.Gardner) and *Cladophora rivularis* (Kuntze) were dominant species in T2; *Diatoma vulgaris*, *Cymbella affinis*, *Navicula phyllepta*, *Fragilaria amphicephaloides*, *Synedra dorsiventralis* (O.Müller), *Gogorevia exilis*, *Mougeotia scalaris* and *Ulothrix zonata* (F.Weber & Mohr) were the dominant species in T3 (Table 1).

The Chao1 index, Shannon index and Evenness index presented dissimilar average values in the different stages (Kruskal–Wallis, *p* < 0.05, Figure 1c). The highest value of the Chao1 index was obtained in T3, and the highest values of the Shannon index and Evenness index were obtained in T2 (Figure 1c).

### 2.2. Niche Analysis

**Figure 2:**
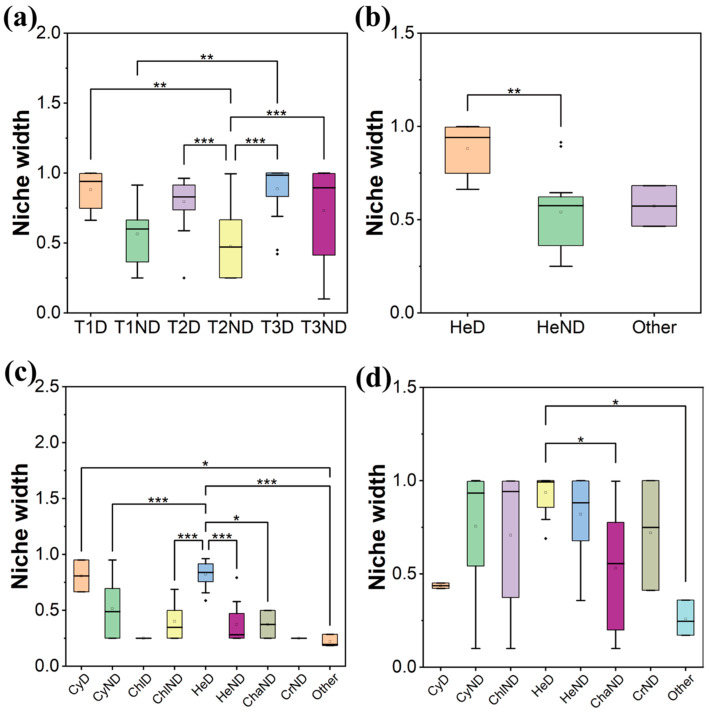
Comparison of niche widths during the colonization of periphytic algal community in the MRP ((**a**) among stages, (**b**) among phyla in T1, (**c**) among phyla in T2, (**d**) among phyla in T3. The x-axis coordinate naming rules are as follows: ‘T1′, ‘T2′ and ‘T3′ indicate three stages; ‘Cy’ indicates Cyanobacteria; ‘Chl’ indicates Chlorophyta; ‘He’ indicates Heterokontophyta; ‘Cha’ indicates Charophyta; ‘Cr’ indicates Cryptista; ‘Other’ indicates species with the relative abundance < 0.1% and the frequency of occurrence < 0.01 in the corresponding stage; ‘D’ indicates dominant species; ‘ND’ indicates non-dominant species. e.g., ‘T1D’ indicates dominant species in the T1 stage, ‘HeND’ indicates non-dominant species of Heterokontophyta). Asterisks indicate the statistical significance (*** *p* < 0.001; ** *p* < 0.01; and * *p* < 0.05).

The niche width showed an overall decreasing and then increasing trend (Figure 2a). The niche widths of the dominant species were always higher than those of the non-dominant species in the three stages; the difference in the average niche width between dominant and non-dominant species first increased (T1 = 0.34 to T2 = 0.38) and then decreased (T2 = 0.38 to T3 = 0.09) (Figure 2a).

The niche width of each phylum showed a decreasing and then increasing trend, which was consistent with the overall trend; the niche widths of Heterokontophyta were always greater than those of other phyla, and the difference in the average niche width between Heterokontophyta and other phyla increased (T1 = 0.31 to T2 = 0.42) and then decreased (T2 = 0.42 to T3 = 0.33) (Figure 2b–d). At the species level, the niche width of *Diatoma vulgaris* was the widest (*Bi* = 0.99) in T1, the niche width of *Fragilaria amphicephaloides* was the widest (*Bi* = 0.84) in T2 and the niche width of *Cymbella affinis* was the widest (*Bi* = 0.98) in T3 (Figure S1). And the niche width and dominance of species showed a significant positive correlation (*p* < 0.05) (Figure S2).

**Figure 3:**
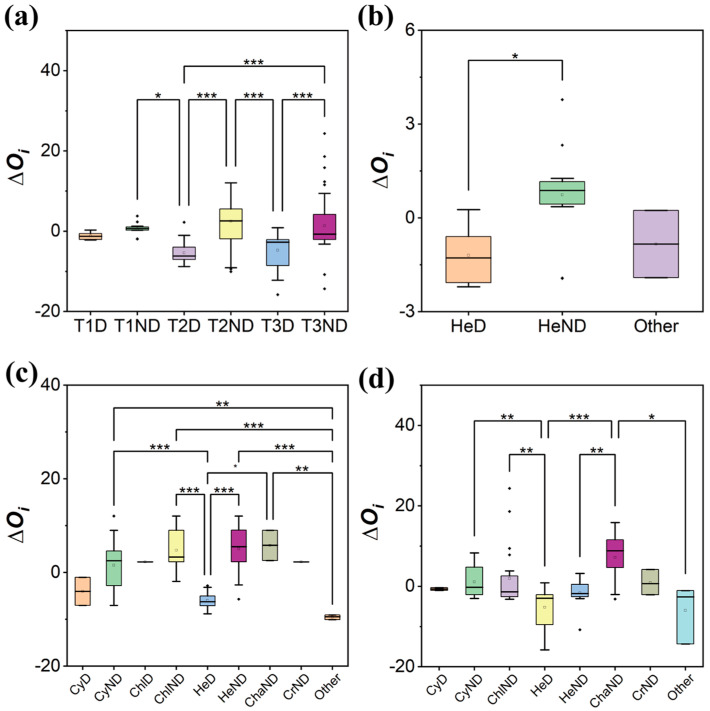
Comparison of ∆*O_i_* during the colonization of periphytic algal community ((**a**) among stages, (**b**) among phyla in T1, (**c**) among phyla in T2, (**d**) among phyla in T3. The x-axis coordinate naming rules are as follows: ‘T1′, ‘T2′ and ‘T3′ indicate three stages; ‘Cy’ indicates Cyanobacteria; ‘Chl’ indicates Chlorophyta; ‘He’ indicates Heterokontophyta; ‘Cha’ indicates Charophyta; ‘Cr’ indicates Cryptista; ‘Other’ indicates species with the relative abundance < 0.1% and the frequency of occurrence < 0.01 in the corresponding stage; ‘D’ indicates dominant species; ‘ND’ indicates non-dominant species; the closer the absolute value of ∆*Oi* was to 0, the lower the niche overlap was. e.g., ‘T1D’ indicates dominant species in the T1 stage, ‘HeND’ indicates non-dominant species of Heterokontophyta). Asterisks indicate the statistical significance (*** *p* < 0.001; ** *p* < 0.01; and * *p* < 0.05).

The ∆*O_i_* showed an overall decreasing and then increasing trend; the ∆*O_i_* values of the dominant species were always smaller than those of the non-dominant species in the three stages, and the difference in the average ∆*O_i_* between dominant and non-dominant species increased (T1 = 1.93 to T2 = 8.74) and then decreased (T2 = 8.74 to T3 = 3.45) (Figure 3a).

The ∆*O_i_* of each phylum showed a decreasing and then increasing trend, consistent with the overall trend; the ∆*O_i_* values of Heterokontophyta were always smaller than those of other phylum, the difference in the average ∆*O_i_* between Heterokontophyta and other phyla increased (T1 = 1.56 to T2 = 4.76) and then decreased (T2 = 4.76 to T3 = 0.88) (Figure 3b–d). At the species level, the absolute value of ∆*O_i_* of *Diatoma vulgaris* was closest to 0 (|∆*O_i_*| = 0.60) in T1, the absolute value of ∆*O_i_* of *Fragilaria amphicephaloides* was closest to 0 (|∆*O_i_*| = 2.24) in T2 and the absolute value of ∆*O_i_* of *Cymbella affinis* was closest to 0 (|∆*O_i_*| = 0.76) in T3 (Figure S1). And the closer the absolute value of ∆*O_i_* was to 0, the lower the niche overlap and the higher the species dominance were (*p* < 0.05) (Figure S2).

**Figure 4:**
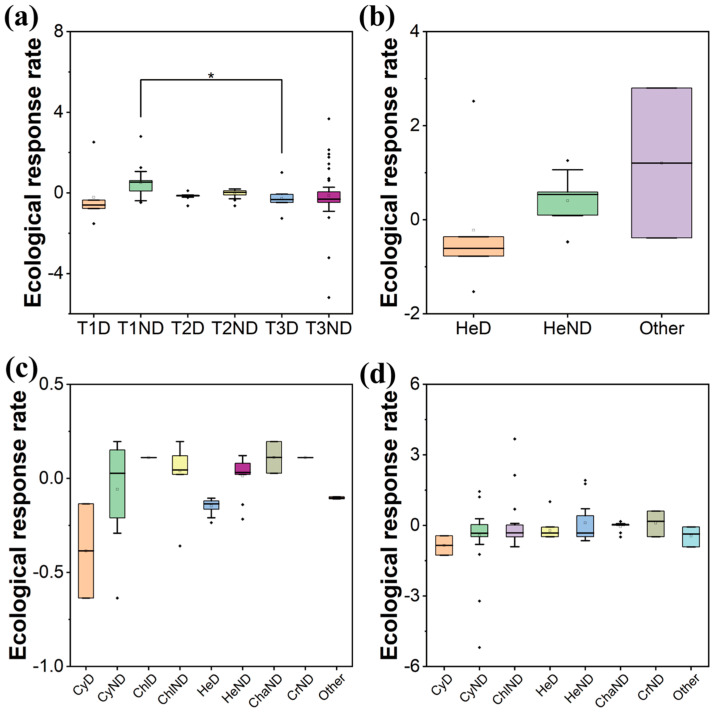
Comparison of ecological response rate during the colonization of periphytic algal community ((**a**) among stages, (**b**) among phyla in T1, (**c**) among phyla in T2, (**d**) among phyla in T3. The x-axis coordinate naming rules are as follows: ‘T1′, ‘T2′ and ‘T3′ indicated three stages; ‘Cy’ indicates Cyanobacteria; ‘Chl’ indicates Chlorophyta; ‘He’ indicates Heterokontophyta; ‘Cha’ indicates Charophyta; ‘Cr’ indicates Cryptista; ‘Other’ indicates species with the relative abundance < 0.1% and the frequency of occurrence < 0.01 in the corresponding stage; ‘D’ indicates dominant species; ‘ND’ indicates non-dominant species. e.g., ‘T1D’ indicates dominant species in the T1 stage, ‘HeND’ indicates non-dominant species of Heterokontophyta). Asterisks indicate the statistical significance (* *p* < 0.05).

The difference in ecological response rate between dominant and non-dominant species showed a decreasing trend (T1 = 0.74 to T3 = 0.15) (Figure 4a). In T1, the ecological response rate was lowest for dominant Heterokontophyta (average value = −0.61) and highest for Other (average value = 1.20). In T2 and T3, dominant Heterokontophyta and Cyanobacteria showed lower response rates (average value = −0.15 and −0.39 in T2; average value = −0.23 and −0.85 in T3), while Chlorophyta and Charophyta had higher rates (average value = 0.11 and 0.11 in T2; average value = 0.01 and 0.10 in T3). Overall, most species exhibited low response rates (Figure 4b–d). As colonization progressed, the response rate decreased across phyla, with the difference between Heterokontophyta and others initially increasing then decreasing (Figure 4b–d). At the species level, the ecological response rate was lower and the species dominance was higher (*p* < 0.05) (Figure S2).

**Figure 5:**
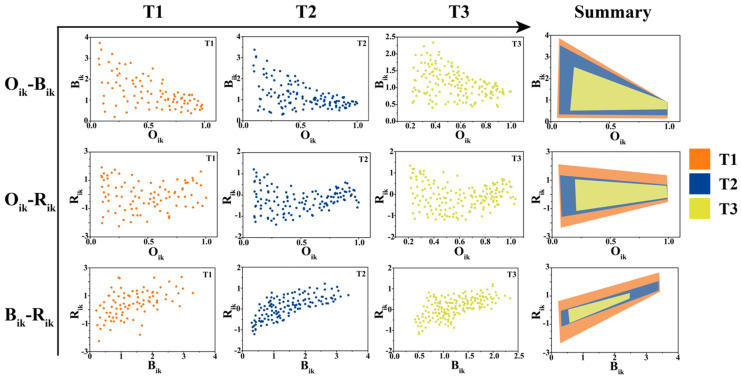
Three-dimensional evaluation framework of niche overlap degree (*O_ik_*), niche width difference (*B_ik_*) and ecological response rate difference (*R_ik_*) during the colonization of periphytic algal community (the scatter diagram shows the relationship between the niche parameters of each stage of the periphytic algae, and the range of the graph in the ‘Summary’ section represents the 95% confidence interval).

Based on the niche analysis data, we constructed an evaluation framework of the differences in niche overlap degree, niche width and ecological response rate. In the colonization process, the range of the niche parameters in the T1 stage in this framework was the largest, and the range of the niche parameters in each subsequent stage did not exceed that of the previous stage (Figure 5).

By comparing the niche between different stages, it was found that the niche width difference (*B_ik_*) among different species decreased, the niche overlap degree among different species (*O_ik_*) increased and the ecological response rate difference (*R_ik_*) remained stable. With the increase in *O_ik_*, the *B_ik_* among different species decreased, and the *R_ik_* remained stable (Figure 5). With the increase in *B_ik_* among different species, the *R_ik_* increased (Figure 5).

**Figure 6:**
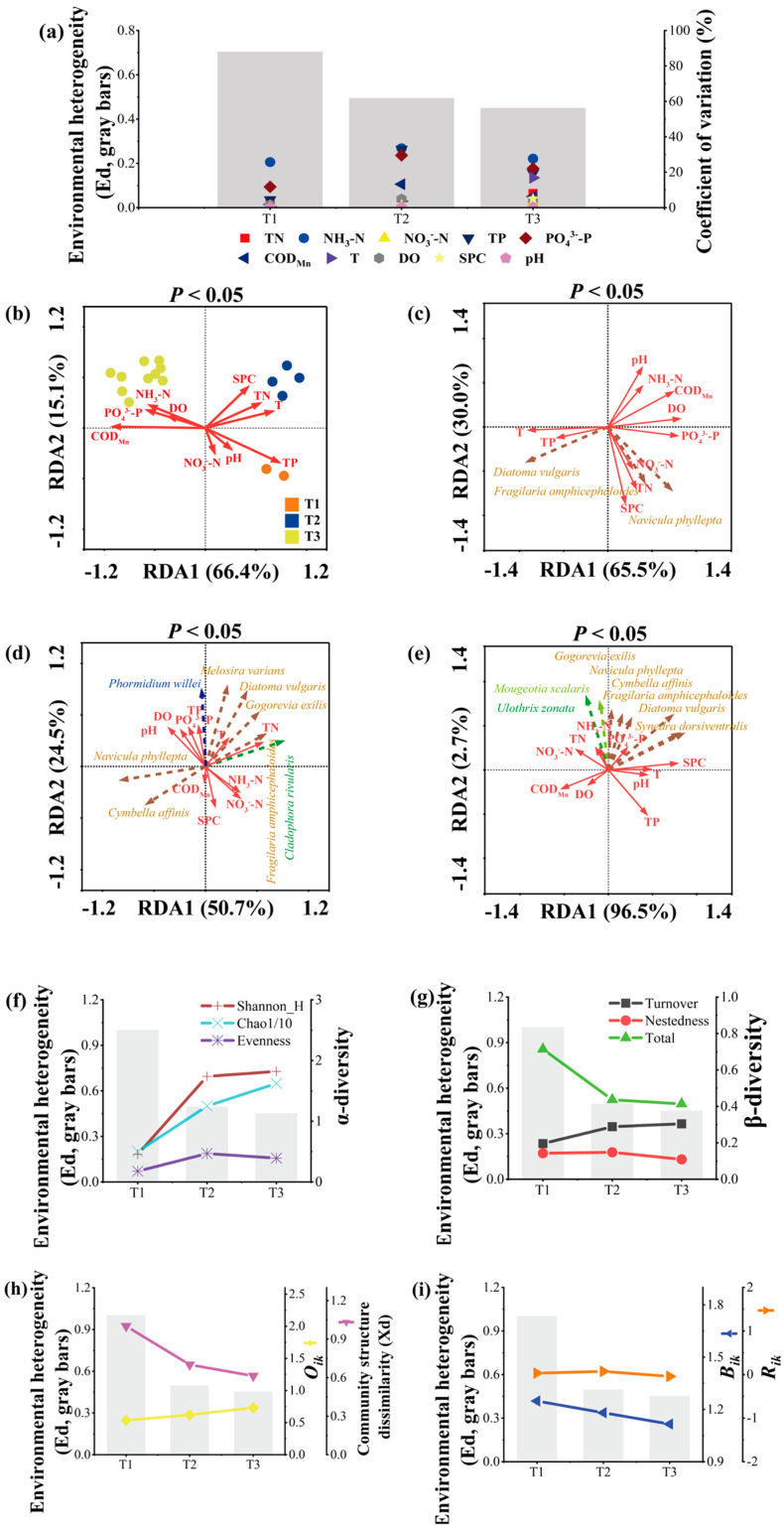
Environmental heterogeneity and community dynamic in different periphytic algal community colonization stages ((**a**) environmental heterogeneity, (**b**) RDA based on community structure and environmental factors (Eigenvalue (axis 1 + aixs 2) = 81.5%, *p* < 0.05), (**c**) RDA based on dominant species abundance and environmental factors in T1 (Eigenvalue (axis 1 + aixs 2) = 95.5%, *p* < 0.05), (**d**) RDA based on dominant species abundance and environmental factors in T2 (Eigenvalue (axis 1 + aixs 2) = 75.2%, *p* < 0.05), (**e**) RDA based on dominant species abundance and environmental factors in T3 (Eigenvalue (axis 1 + aixs 2) = 99.2%, *p* < 0.05), (**f**) α-diversity, (**g**) β-diversity, (**h**) niche overlap degree among different species (*O_ik_*) and community structure dissimilarity (Xd), (**i**) niche width difference (*B_ik_*) and ecological response rate difference (*R_ik_*)).

The value of environmental factors, the coefficient of variation (CV%) of environmental factors and the average environmental dissimilarity (Ed) were not significantly different between the stages (*p* > 0.05) (Table 2 and Figure 6a). The arrangement of community structure in the RDA was mainly related to the colonization stage; the first two axes accounted for 81.5% of the variance (axis 1: 66.4%; axis 2: 15.1%), with T, pH, DO, SPC, COD_Mn_ and nutrients (TN, NO_3_^-^-N, NH_3_-N, TP, PO_4_^3-^-P) being the main explanatory variables (*p* < 0.01) (Figure 6b). Community structure from T1 was grouped together by the lower right of the graph and was positively related to TP. Community structure from T2 was grouped together by the upper right of the graph and was positively related to T and TN. Community structure from T3 was grouped together by the upper left of the graph and was positively related to NH_3_-N and PO_4_^3−^-P (Figure 6b).

In T1, dominant species abundances were positively related to TP and NO_3_^−^-N. In T2, dominant species abundances were significantly related to T and TN. In T3, dominant species abundances were significantly related to NH_3_-N and PO_4_^3−^-P (Figure 6c–e).

In the colonization process of the periphytic algal community, the α-diversity was increased, the total value of β-diversity was decreased, while turnover increased and nestedness decreased (Figure 6f,g). The community structure dissimilarity (Xd) decreased (Figure 6h). The niche width difference (*B_ik_*) among different species decreased, the niche overlap degree among different species (*O_ik_*) increased and the ecological response rate difference (*R_ik_*) remained stable (Figure 6h,i).

## 3. Discussion

### 3.1. Colonization Process and the Influencing Factors

It was previously reported that the colonization process of periphytic algal communities can be classified into three distinct stages: the initial colonization stage, the community formation stage, and the primary succession stage [9,23]. The findings of our study align with these findings, revealing the same three stages. However, there are some subtle differences in the characteristics of each stage.

The initial colonization stage in the MRP took 3–6 days, which was longer than in other waters (less than or equal to 1 day) [24,25]. This is attributed to the lower surface roughness and higher specific surface area of the concrete substrates in the MRP, making it more difficult for periphytic algae to adhere through electrostatic interaction and physical adhesion. Consequently, this delays the completion of the initial colonization stage [26,27]. The α-diversity in this stage was lower than in other waters, indicating a reduced number of species participating in the colonization process [23,27]. Unlike natural waters, where pioneer species are typically Cyanobacteria and Heterokontophyta, only Heterokontophyta served as the pioneer species in the MRP due to the specific characteristics of the concrete substrate and water velocity, which are more conducive to Heterokontophyta growth [28,29,30]. The niche width was larger and the niche overlap degree was smaller in this stage, indicating that more resources could be available for periphytic algae [17], which indicated the MRP habitats had sufficient capacity for algal colonization. In the initial colonization stage, the concrete channel habitat of the MRP delayed the completion of this stage, reducing the number of periphytic algal species and allowing Heterokontophyta to be the pioneer species.

The community formation stage in the MRP was shorter than in other water bodies [24,25]. This is because, in the absence of other aquatic organisms, periphytic algae do not have to compete with others for nutrients and light, allowing for faster proliferation and community formation [31,32]. Although the nutrient content of the MRP was low, the river-like continuous flow provided sufficient nutrients for the growth of periphytic algae, meeting the nutritional requirements for their rapid growth during this stage [33,34]. The increase in α-diversity compared to the initial colonization stage suggests the involvement of new species in community formation. Given the relatively closed system characteristics of the MRP, these new species likely originated from the migration of flowing water or entry from the air [35,36]. Compared with the initial colonization stage, the algal niche width decreased and the niche overlap degree increased in the community formation stage, indicating that with the increase in species, the resources available for algae decreased [17], the competition or sharing of resources among species increased and intense competition was observed. In the community formation stage, habitats of concrete channels excluded competition for resources from other aquatic organisms for periphytic algae, and the flowing water provided sufficient nutrients and new algae to facilitate the completion of this stage; the capacity of the habitat for periphytic algae was close to the limit.

The cell density in the primary succession stage was higher than in other waters [24,25]. This is because the total number of consumers feeding on periphytic algae was lower in the MRP than in other waters. Additionally, there were no other stress conditions in the MRP slope area, and less consumer feeding and competition from other producers maximized the environmental capacity for periphytic algae [37,38]. The α-diversity of periphytic algae in this stage was lower than in other waters, indicating that fewer species were able to enter the MRP habitat and participate in primary succession. The concrete channel consistently isolated exotic algae [23,27]. Compared to the community formation stage, the differences in niches among different species decreased in this stage, indicating that species’ abilities to utilize resources gradually converged and competition among species intensified with the onset of primary succession [17,30]. The increase in niche width and decrease in the niche overlap degree of dominant species in this stage suggest that the dominant species resulting from interspecific competition were able to monopolize and utilize resources more efficiently [17,28]. In the primary succession stage, the unique habitat of the MRP provided a high capacity for periphytic algae, but the community exhibited low biodiversity and increased interspecific competition, resulting in dominant species that were able to exclusively and more efficiently utilize resources.

The community was primarily influenced by total phosphorus during the initial colonization stage in our study; this was due to the reduced biomass limit of initially colonizing periphytic algae from a lower phosphorus source, hindering timely substrate improvement and colonization conditions for subsequent algae, thus delaying the completion of the initial colonization stage [39,40]. The community was mainly affected by temperature and total nitrogen in the community formation stage. This was due to the fact that the algae in the community had already adapted to the channel environment in this stage, the diversity and cell density increased faster, the interspecific competition intensified, the competition for resources became more intense and the species that were able to take more nitrogen and adapt to the temperature of the channel were more able to dominate and change the community structure [40,41]. During the primary succession stage, the community structure and dominant species of the periphytic algae were mainly affected by ammonia nitrogen and orthophosphate, because the diversity and biomass reached the highest values at this stage, the sharing and competition for resources among species were more refined and ammonia nitrogen and orthophosphate, which were easy to be absorbed, became an important target resource [42,43]. It could be said that the changes in the categories of influencing factors could reflect the dynamics of environmental filtration and interspecific competition on the community structure during the colonization process of periphytic algae.

### 3.2. The Interspecific Competition Driving the Colonization Process

During the initial colonization stage, the limited number of species and low cell density enabled periphytic algae to expand their niche width with minimal niche overlap; this finding follows past studies showing ample resource availability for each species and no intense interspecific competition [17,44]. As the colonization progressed, the number of species increased but the niche overlap was still low, the species composition and dominant species remained relatively stable and niche overlap is known to be positively correlated with the intensity of interspecific competition, indicating that under resource-rich conditions, even if the number of species increased, the weak interspecific competition for resources had minimal impact on the community [45,46]. It has been demonstrated that Heterokontophyta, owing to their distinct attachment form and cell morphology, possess higher attachment stability and lower resistance to water flow, making them more suitable for colonizing flat substrate surfaces at higher flow rates than Cyanobacteria, Chlorophyta, Charophyta and Cryptista, in combination with changes in community structure during this phase, demonstrating that environmental adaptation drove the emergence of dominant species [47]. Therefore, due to resource abundance, the weak interspecific competition during the initial colonization stage had little effect on the community, and the ability to adapt to the environment drove the emergence of dominant species.

During the community formation stage, the species richness and niche overlap both increased, indicating that the entry of a large number of species led to intense competition for resources under the limited resource conditions, which is in line with existing studies related to ecological niche theory [48,49]. The dominant species exhibited low niche overlap, indicating that the specific species showed no intense interspecific competition with others [17]. However, the niche overlap of dominant species increased alongside the community formation time, in contrast to the niche overlap of planktonic algae in similar studies, suggesting that under conditions of resource scarcity, dominant species in a community of periphytic algae need to participate in more intense interspecific competition [44,50]. The dominant species’ niche widths decreased, suggesting that dominant species were losing their strong environmental adaptation ability [28]. The rise in α-diversity in the community suggested that moderate interspecific competition could promote biodiversity [51]. It was found that the interspecific competition increased in this stage.

During the primary succession, the niche overlap increased, meaning the interspecific competition intensified [42,52]. As the primary succession progressed, the number of species was relatively stable and the niche overlap decreased, but the difference in niche overlap between dominant and non-dominant species increased, suggesting that the dominant species were still expanding their dominance over the non-dominant species in the intensified competition [53]. On the other hand, the number of dominant species decreased and the species variety changed, indicating that, similar to other algal communities, the dominant species in the periphytic algal community established their dominance through competition [54]. At the same time, the niche width of the dominant species increased and the ecological response rate decreased, indicating that the dominant species obtained more resources through interspecific competition and enhanced their own environmental adaptive capacity [45,46]. Therefore, in the primary successional stage, interspecific competition further intensified, and the dominant species consolidated its dominant position through interspecific competition and the resources’ obtainability.

The β-diversity was primarily determined by two components, turnover and nestedness, which result from species replacement between different species and changes in species richness [55,56]. In the colonization process, the turnover contribution was higher than the nestedness contribution in the β-diversity, indicating that species replacement was the main factor influencing the differences in community structure during colonization [57]. This phenomenon might be attributed to the competitive winning species continuously replacing competitive losing species during colonization, and as interspecific competition intensified, the number of species replaced through interspecific competition increased, and the impact on community structure was enhanced [58]. Therefore, interspecific competition leading to species replacement was the primary influence shaping periphytic algal communities.

## 4. Materials and Methods

### 4.1. Sample Station and Sample Collection

The study site was located in the Shahe aqueduct of the Middle Route of the South to North Water Diversion Project (MRP) (33.70° N, 112.94° E). This study site is an important component of the MRP, and it is a representative concrete channel habitat. Based on our previous investigations of the MRP and the objectives of support funding, the experiment was timed from 15 April 2019 to 30 May 2019 in the spring, when the growth of the periphytic algae is most rapid and has the greatest impact on water conveyance in the MRP. The in situ periphytic algal colonization investigation was conducted in the MRP using artificial concrete substrates, which was the same material of the MRP. At the start of the experiment, 120 clean concrete artificial substrates (20 × 20 cm) were deployed on side slopes of the MRP at 0.35 m depth below the water surface. Periphytic algal samples and water samples were collected every three days.

Periphytic algae pieces were removed from the artificial substrate using a spade and divided into four 10 cm² pieces with a knife. Each small piece was placed into a white porcelain dish and stirred evenly with 100 mL of pure water. Samples were collected every 3 days, with four 10 cm² algal samples serving as duplicate samples each time. These duplicate samples were stored at 4 °C for species identification and cell density counting.

Water samples were collected synchronously for chemical analysis. Water samples (1 L) were collected at 0.35 m depth using a water collector and poured into sampling bottles to be stored at 4 °C before test.

### 4.2. Periphytic Algal Identification and Quantity

Each 10 cm² periphytic algal sample was individually filled into a 50 mL sample bottle and fixed by adding Lugol’s solution after being diluted with pure water to 50 mL. The analysis of taxonomic composition and abundance of each sample was divided into Heterokontophyta and other phyla (including Cyanobacteria, Chlorophyta, Charophyta and Cryptista).

Heterokontophyta: Periphytic algal samples were treated with concentrated nitric acid (HNO_3_) to clean organic matter that causes disturbance, according to the technical guidelines for water ecological monitoring–aquatic organism monitoring and evaluation of rivers (on trial) [59]. Cleaned samples were made into permanent slides, also according to the technical guidelines for water ecological monitoring–aquatic organism monitoring and evaluation of rivers (on trial) [59]. Permanent slides were examined with a light microscope (Olympus CX23 microscope, Tokyo, Japan) at a magnification of 1000×. The identification and enumeration of diatoms were performed according to the technical guidelines for water ecological monitoring–aquatic organism monitoring and evaluation of rivers (on trial) [59]. At least 400 cells were counted with a counting chamber and determined to the species level for each permanent slide. For the determination and nomenclature of Heterokontophyta, the identification monographs of Hu and Wei [60] and *AlgaeBase* [61] were used.

Other phyla: The taxonomic composition and abundance of other algae were also analyzed. At least 50 visual fields at 400× magnification were surveyed, and all algae were counted with a counting chamber and determined to the species level. For the determination and nomenclature of other algae, the identification monographs of Hu and Wei [60] and *AlgaeBase* [61] were used.

### 4.3. Environmental Parameters

Water temperature (T), pH, turbidity (Tur), specific conductivity (SPC) and dissolved oxygen (DO) were monitored by YSI (proplus, Xylem, Washington, DC, USA), and flow velocity (V) was monitored by a flowmeter (Flowatch, Geneva, Switzerland). Other environmental parameters of the water samples such as total nitrogen (TN), nitrate nitrogen (NO_3_^−^-N), ammonium nitrogen (NH_3_-N), total phosphorus (TP), orthophosphate (PO_4_^3−^-P) and potassium permanganate index (COD_Mn_) were determined according to APHA [62].

### 4.4. Niche Analysis

The dominance of dominant periphytic algal species $D_{i}$ was calculated as follows:

(1) $$D_{i}=\frac{n_{i}}{N}\cdot f_{i}$$

where $n_{i}$ = the number of algal species *i* in the sample, N = the total number of all algae individuals in the sample and $f_{i}$ = the frequency of occurrence of algal species *i* in samples.

Niche width $B_{i}$ was calculated as follows:

(2) $$P_{ij}=\frac{n_{ij}}{N_{i}}$$

(3) $$B_{i}=\frac{1}{r\cdot \sum_{j=1}^{r}{\left(P_{ij}\right)}^{2}}$$

(4) $$B_{ik}=\frac{B_{i}}{B_{k}}$$

where $n_{ij}$ = the number of algal species *i* in the sample *j*, $N_{i}$ = the total number of algal species *i* in the all samples and r = the total number of samples. $B_{ik}$ = the difference between the niche width of species *i* and the niche width of species *k*.

The niche overlap degree $O_{ik}$ was calculated as follows:

(5) $$O_{ik}=\frac{\sum_{j=1}^{r}\left(P_{ij}P_{kj}\right)}{\sum_{j=1}^{r}{{(P}_{ij})}^{2}\sum_{j=1}^{r}{\left(P_{kj}\right)}^{2}}$$

(6) $${∆O}_{i}=\sum_{k=1}^{n}O_{ik}-\sum_{i=1}^{m}O_{ik}$$

(7) $${∆O}_{ik}=\frac{{∆O}_{i}}{{∆O}_{k}}$$

where $P_{kj}$ represents the number of species *k* in the sample *j*, $\sum_{k=1}^{n}O_{ik}$ denotes the total number of resources occupied by species *i* from other species and $\sum_{i=1}^{m}O_{ik}$ represents resources of species *i* occupied by other species, where *i* = *k*, ${∆O}_{i}$ indicates the resource occupancy dynamics of species *i*.

The ecological response rate $R_{i}$ considers the ecological responses of species with different niche widths to the same environmental conditions; it was calculated as follows:

(8) $$R_{i}=\frac{B_{i}}{∆O}$$

(9) $$R_{ik}=\frac{R_{i}}{R_{k}}$$

where $R_{ik}$ = the different between ecological response rate of species *i* and ecological response rate of species *k*.

### 4.5. Environmental Heterogeneity

Environmental heterogeneity was estimated by computing the average dissimilarity between sites based on environmental factors [63]. For each colonization stage, we computed a Euclidean distance matrix (*Vegan* package version 2.6-6.1, R) and calculated the dissimilarity between sites (*Ed*) as follows:

(10) $$Ed=\left(\frac{Euc}{{Euc}_{max}}\right)+0.001$$

where *Euc* is the Euclidean distance between two sites and *Euc_max_* corresponds to the maximum Euclidean distance considering all the pairwise distances in the overall dataset. Here, 0.001 was added to account for zero similarity between sites [64]. Then, we calculated the mean Ed of each computed similarity matrix and used it as an index of environmental heterogeneity in colonization stages. We compared the mean of ranked dissimilarities between stages to the mean of ranked dissimilarities within phases with an analysis of similarities (ANOSIM). In addition, we calculated the coefficient of variation (CV%) for each variable and hydrological phase as the standard deviation divided by the mean of each variable.

To test whether the environmental conditions in stages sites differed significantly, we performed permutational multivariate analysis of variance (PERMANOVA). We first tested for homogeneity of multivariate dispersion (PERMDISP) with the *betadisper* function (*Vegan* package, R), which compares the within-group spread among groups using the average value of the individual observation distances to the centroid of one’s own group. Differences in environmental conditions were also analyzed with a performed permutational multivariate analysis of variance (PERMANOVA) to test the effects of stages (*adonis* function in *Vegan* package, R) [64].

### 4.6. Periphytic Algal Community Structure

We computed a Bray–Curtis matrix on the basis of abundances for each stage to calculate the dissimilarity (Xd) between the local communities as follows:

(11) $$Xd=\left(\frac{Bray}{{Bray}_{max}}\right)+0.001$$

where *Bray* is the Bray–Curtis dissimilarity between two communities and *Bray_max_* corresponds to the maximum Bray–Curtis dissimilarity considering the overall dataset. Then, we calculated the mean Xd of each matrix computed, which was used as a value of community turnover.

To test whether the community structure in the stages differed significantly, we performed a permutational multivariate analysis of variance (PERMANOVA). Since PERMDISP was significant, we ran the PERMANOVA with the *betadisper* function (*Vegan* package, R). We formed taxonomically based β- diversity matrices, in which total β-diversity was partitioned into turnover and nestedness components based on presence–absence data using the *beta.pair* function in the R package *betapart* (version 1.6) [65]. To visualize the taxonomic similarity across local communities, Principal Coordinates Analysis (PCoA) was performed with the R package *Vegan* [66].

### 4.7. Statistical Analysis

The cell density, environmental parameters and alpha diversity indexes charts were drawn with Origin version 9.0. Niche analysis was performed with the “spaa” package (version 0.2.2) in R 4.0.5. Redundancy analysis (RDA) was performed using CANOCO 4.5.

## 5. Conclusions

Based on niche analysis, we uncovered the interspecific competition of periphytic algal community colonization and its driving force in the world’s largest water diversion project; the colonization process of the periphytic algal community was divided into three stages: the initial colonization stage, community formation stage and primary succession stage. In the initial colonization stage with Heterokontophyta as the first phylum, habitats had sufficient capacity for algae and interspecific competition was moderated. In the community formation stage, though α-diversity rose, interspecific competition increased. After community formation, interspecific competition was further intensified in the primary succession stage. Based on the niche analysis, it was found that periphytic algae with wider niche widths, lower niche overlap and lower ecological response rates had higher dominance ability. It could be concluded that interspecific competition was increased during the colonization process, which led to species replacement and shaped the periphytic algal communities.

## Figures and Tables

**Table 1:** Algal species in different stages. In the first column of this table, ‘Sobs’ is the number of species observed. In the third column of this table, species with the relative abundance < 0.1% and the frequency of occurrence < 0.01 in the corresponding stage are classified in the ‘other’ classification, and ‘n = number’ indicates the number of species included in ‘Other’. In the fourth column of this table, ‘D’ indicates dominant species and ‘ND’ indicates non-dominant species.

Stage	Phylum	Species	Dominant Species
T1 (Sobs = 27)	Heterokontophyta	*Diatoma vulgaris*	D
	Heterokontophyta	*Fragilaria amphicephaloides*	D
	Heterokontophyta	*Navicula phyllepta*	D
	Heterokontophyta	*Gogorevia exilis*	ND
	Heterokontophyta	*Cymbella affinis*	ND
	Heterokontophyta	*Synedra dorsiventralis*	ND
		Other (n = 21)	ND
T2 (Sobs = 87)	Cyanobacteria	*Phormidium willei*	D
	Cyanobacteria	*Leibleinia gracilis*	ND
	Cyanobacteria	*Nodularia spumigena*	ND
	Cyanobacteria	*Phormidium allorgei*	ND
	Chlorophyta	*Cladophora rivularis*	D
	Chlorophyta	*Monoraphidium contortum*	ND
	Chlorophyta	*Pseudopediastrum boryanum*	ND
	Heterokontophyta	*Gogorevia exilis*	D
	Heterokontophyta	*Cymbella affinis*	D
	Heterokontophyta	*Diatoma vulgaris*	D
	Heterokontophyta	*Fragilaria amphicephaloides*	D
	Heterokontophyta	*Melosira varians*	D
	Heterokontophyta	*Navicula phyllepta*	D
	Heterokontophyta	*Cyclotella catenata*	ND
	Heterokontophyta	*Gomphonema constrictum*	ND
	Heterokontophyta	*Nitzschia palea*	ND
	Heterokontophyta	*Synedra dorsiventralis*	ND
	Cryptista	*Cryptomonas erosa*	ND
		Other (n = 69)	ND
T3 (Sobs = 131)	Cyanobacteria	*Aphanothece castagnei*	ND
	Cyanobacteria	*Leibleinia gracilis*	ND
	Cyanobacteria	*Merismopedia tranquilla*	ND
	Cyanobacteria	*Nodularia spumigena*	ND
	Cyanobacteria	*Nostoc verrucosum*	ND
	Cyanobacteria	*Phormidium allorgei*	ND
	Cyanobacteria	*Synechococcus ambiguus*	ND
	Cyanobacteria	*Tychonema bornetii*	ND
	Chlorophyta	*Ulothrix zonata*	D
	Chlorophyta	*Monoraphidium contortum*	D
	Chlorophyta	*Lobochlamys segnis*	ND
	Chlorophyta	*Pandorina morum*	ND
	Chlorophyta	*Pseudopediastrum boryanum*	ND
	Chlorophyta	*Scenedesmus quadricauda*	ND
	Heterokontophyta	*Gogorevia exilis*	ND
	Heterokontophyta	*Cymbella affinis*	ND
	Heterokontophyta	*Diatoma vulgaris*	ND
	Heterokontophyta	*Fragilaria amphicephaloides*	D
	Heterokontophyta	*Navicula phyllepta*	D
	Heterokontophyta	*Synedra dorsiventralis*	D
	Heterokontophyta	*Cyclotella catenata*	D
	Heterokontophyta	*Gomphonema constrictum*	D
	Heterokontophyta	*Melosira varians*	D
	Heterokontophyta	*Nitzschia palea*	ND
	Charophyta	*Mougeotia scalaris*	ND
	Charophyta	*Spirogyra crassispina*	ND
	Charophyta	*Staurastrum indentatum*	ND
	Cryptista	*Cryptomonas erosa*	ND
		Other (n = 103)	ND

**Table 2:** Habitat data by stage. Data are means ± SD. Different letters denote means that differ from each other with statistical significance *p* < 0.05 (post hoc Tukey’s test).

Stage	T1	T2	T3
TN	1.18 ± 0.04 ^a^	1.12 ± 0.03 ^a^	1.10 ± 0.09 ^a^
NH_3_	0.06 ± 0.01 ^a^	0.04 ± 0.01 ^a^	0.06 ± 0.02 ^a^
NO_3_^−^-N	1.09 ± 0.02 ^a^	1.05 ± 0.02 ^a^	1.05 ± 0.03 ^a^
TP	0.03 ± 0.01 ^a^	0.02 ± 0.01 ^a^	0.01 ± 0.00 ^a^
PO_4_^3−^-P	0.01 ± 0.00 ^a^	0.01 ± 0.00 ^a^	0.01 ± 0.00 ^a^
COD_Mn_	1.63 ± 0.33 ^a^	1.91 ± 0.25 ^a^	2.06 ± 0.14 ^a^
T	23.67 ± 2.14 ^a^	24.96 ± 0.58 ^a^	25.73 ± 0.11 ^a^
DO	11.52 ± 0.18 ^a^	11.39 ± 0.55 ^a^	11.48 ± 0.49 ^a^
SPC	217.95 ± 1.48 ^a^	220.84 ± 3.61 ^a^	211.13 ± 10.61 ^a^
pH	8.42 ± 0.01 ^a^	8.44 ± 0.01 ^a^	8.42 ± 0.01 ^a^

## Data Availability

The original contributions presented in the study are included in the article/Supplementary Materials; further inquiries can be directed to the corresponding author.

## Supplementary Materials

The following supporting information can be downloaded at: https://www.mdpi.com/article/10.3390/plants13152067/s1, Figure S1. The dominance, niche width, niche overlap, Δ*Oi* and ecological response rate in colonization process ((a) T1 stage, (b) T2 stage, (c) T3 stage). sp1-*Phormidium allorgei*, sp2-*Nodularia spumigena*, sp3-*Phormidium willei*, sp4-*Tychonema bornetii*, sp5-*Nostoc verrucosum*, sp6-*Leibleinia gracilis*, sp7-*Merismopedia tranquilla*, sp8-*Synechococcus ambiguus*, sp9-*Aphanothece castagnei*, sp10-*Lobochlamys segnis*, sp11-*Pseudopediastrum boryanum*, sp12-*Cladophora rivularis*, sp13-*Monoraphidium contortum*, sp14-*Scenedesmus quadricauda*, sp15-*Mougeotia scalaris*, sp16-*Staurastrum indentatum*, sp17-*Pandorina morum*, sp18-*Ulothrix zonata*, sp19-*Spirogyra crassispina*, sp20-*Diatoma vulgaris*, sp21-*Cymbella affinis*, sp22-*Navicula phyllepta*, sp23-*Fragilaria amphicephaloides*, sp24-*Synedra dorsiventralis*, sp25-*Gogorevia exilis*, sp26-*Melosira varians*, sp27-*Nitzschia palea*, sp28-*Gomphonema constrictum*, sp29-*Cyclotella catenata*, sp30-*Cryptomonas erosa*, sp31-Other). Figure S2. The relationships between dominance and niche ((a) dominance and niche width, (b) dominance and Δ*Oi*, (c) dominance and ecological response rate). Table S1. Species list that observed in this study.
