# Supplementary material for: Competition in the Periphytic Algal Community during the Colonization Process: Evidence from the World’s Largest Water Diversion Project
Source: Plants (Basel). 2024 Jul 26;13(15):2067. doi: 10.3390/plants13152067 (PMC11314427; doi:10.3390/plants13152067)
Supplement: Supplementary file 1 [file plants-13-02067-s001.zip › plants-3085081-supplementary.pdf]

Supporting information

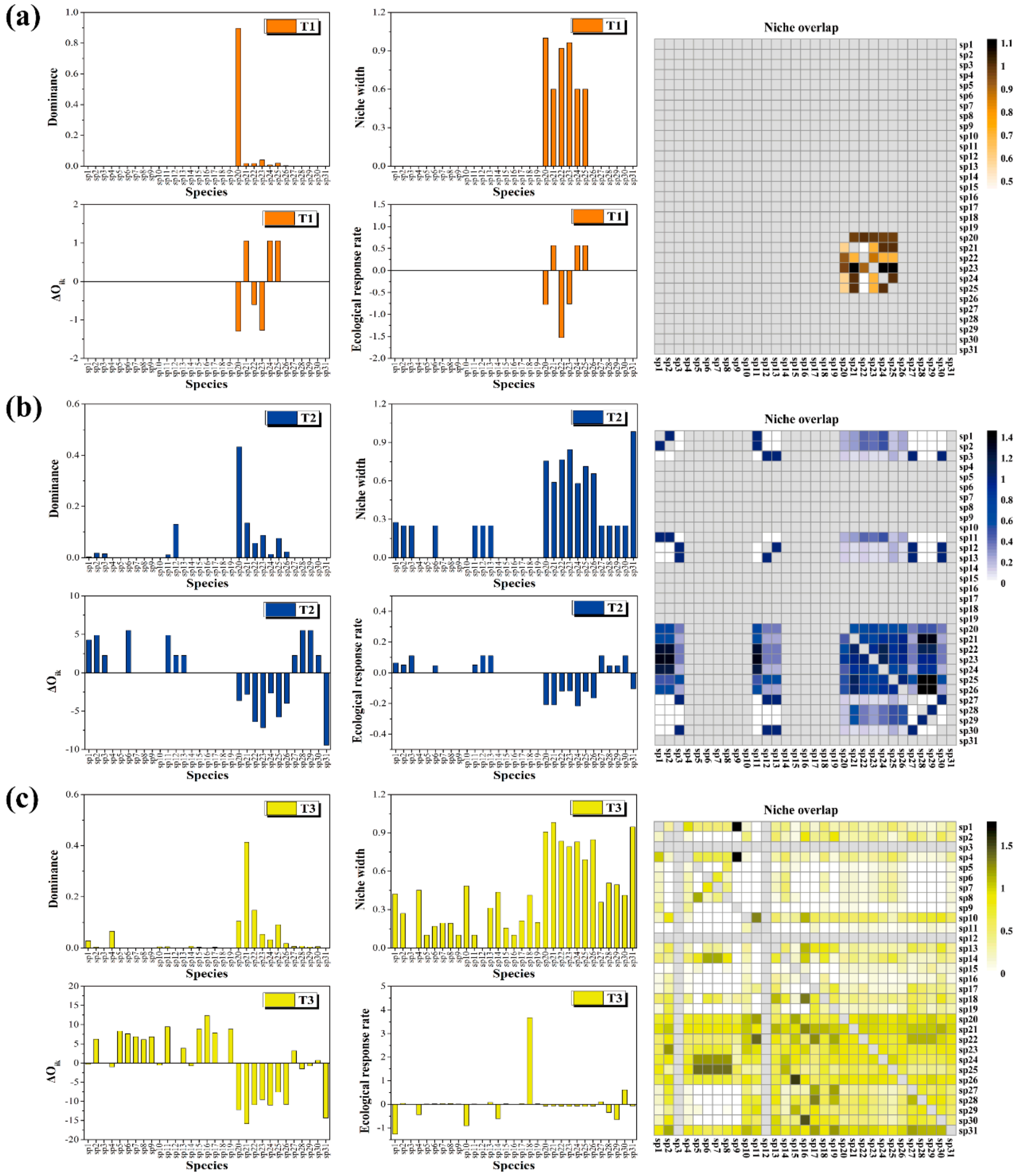

Figure S1. The dominance, niche width, niche overlap, ΔO_i and ecological response rate in colonization process ((a) T1 stage, (b) T2 stage, (c) T3 stage). sp1−*Phormidium allorgei*, sp2-*Nodularia spumigena*, sp3-*Phormidium willei*, sp4-*Tychonema bornetii*, sp5-*Nostoc verrucosum*, sp6-*Leibleinia gracilis*, sp7-*Merismopedia tranquilla*, sp8-*Synechococcus ambiguus*, sp9-*Aphanothece castagnei*, sp10-*Lobochlamys segnis*, sp11-*Pseudopediastrum boryanum*, sp12-*Cladophora rivularis*, sp13-*Monoraphidium contortum*, sp14-*Scenedesmus quadricauda*, sp15-*Mougeotia scalaris*, sp16-*Staurastrum indentatum*, sp17-*Pandorina morum*, sp18-*Ulothrix zonata*, sp19-*Spirogyra crassispina*, sp20-*Diatoma vulgaris*, sp21-*Cymbella affinis*, sp22-*Navicula phyllepta*, sp23-*Fragilaria amphicephaloides*, sp24-*Synedra dorsiventralis*, sp25-*Gogorevia exilis*, sp26-*Melosira varians*, sp27-*Nitzschia palea*, sp28-*Gomphonema constrictum*, sp29-*Cyclotella catenata*, sp30-*Cryptomonas erosa*, sp31-Other).

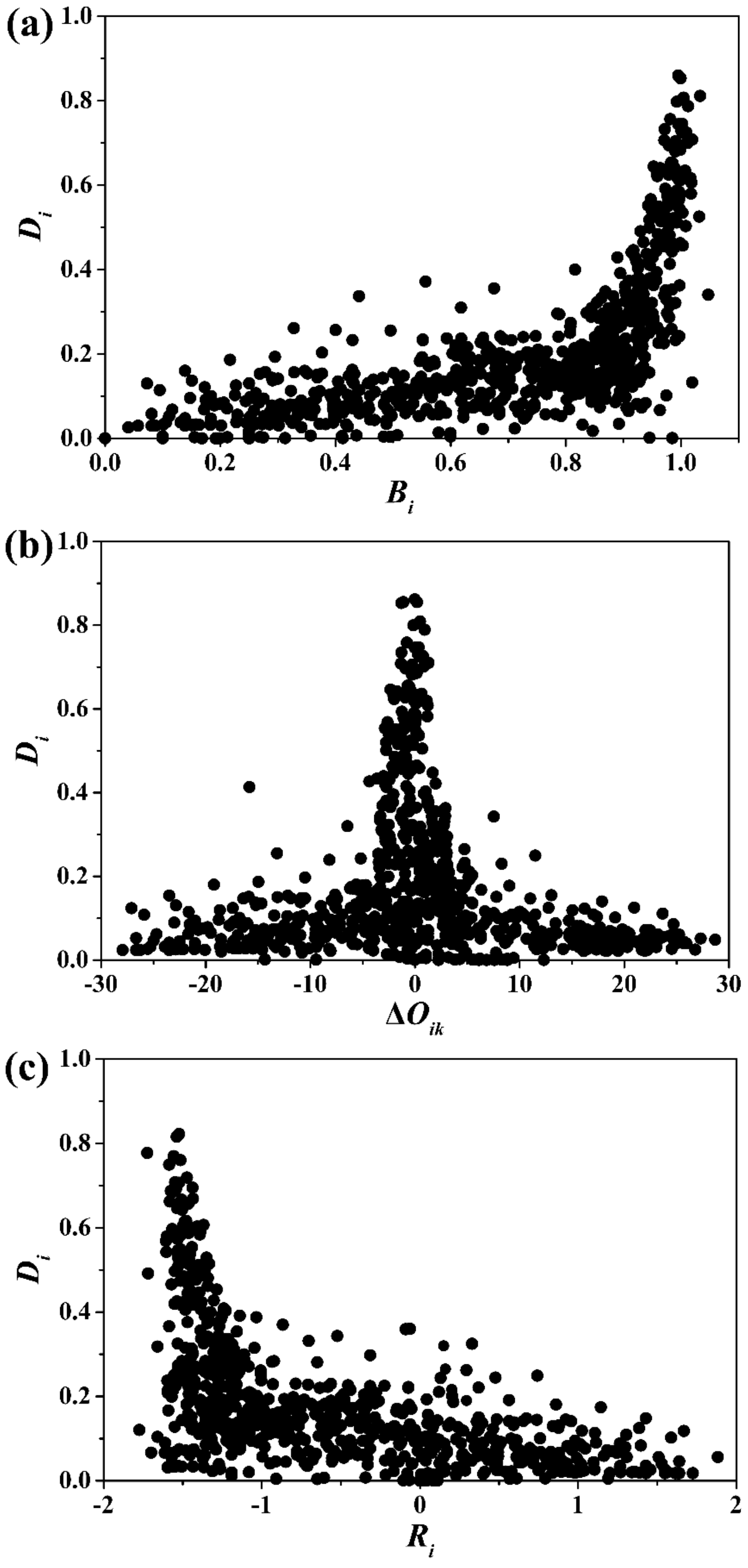

Figure S2. The relationships between dominance and niche ((a) dominance and niche width, (b) dominance and ΔO_i, (c) dominance and ecological response rate).

Table S1. Species list that observed in this study.

Species name
Achnanthidium affine (Grunow) Czarnecki 1994
Actinastrum hantzschii Lagerheim 1882
Adlafia minuscula (Grunow) Lange-Bertalot 1999
Aneumastus tusculus (Ehrenberg) D.G.Mann & A.J.Stickle 1990
Aphanothece castagnei (Kützing) Rabenhorst 1865
Asterionella formosa Hassall 1850
Asterochloris glomerata (Warén) Skaloud & Peksa 2010
Aulacoseira ambigua (Grunow) Simonsen 1979
Caloneis alpestris (Grunow) Cleve 1894
Cavinula lapidosa (Krasske) Lange-Bertalot 1996
Chaetophora lobata Schrank 1783
Chamaepinnularia begeri (Krasske) Lange-Bertalot 1996
Chroococcus minor (Kützing) Nägeli 1849
Cladophora fracta (O.F.Müller ex Vahl) Kützing 1843
Cladophora rivularis (Linnaeus) Kuntze 1891
Closteriopsis longissima (Lemmermann) Lemmermann 1899
Closterium acerosum Ehrenberg ex Ralfs 1848
Cocconeis lineata Ehrenberg 1849
Cocconeis placentula Ehrenberg 1838
Cosmarium formosulum Hoff 1888
Craticula accomoda (Hustedt) D.G.Mann 1990
Cryptomonas erosa Ehrenberg 1832
Ctenocladus circinnatus Borzì 1883
Cyclotella catenata (Brun) H.Bachmann 1911
Cylindrotheca closterium (Ehrenberg) Reimann & J.C.Lewin 1964
Cymatopleura elliptica (Brébisson) W.Smith 1851
Cymbella affinis Kützing 1844
Cymbopleura amphicephala (Nägeli ex Kützing) Krammer 2003
Cymbopleura hybrida (Grunow ex Cleve) Krammer 2003
Cymbopleura lapponica (Grunow ex Cleve) Krammer 2003
Cymbopleura lata (Grunow ex Cleve) Krammer 2003
Cymbopleura naviculiformis (Auerswald ex Heiberg) Krammer 2003
Dactylococcopsis rhaphidioides Hansgirg 1888
Delicata delicatula (Kützing) Krammer 2003
Desmodesmus abundans (Kirchner) E.H.Hegewald 2000
Diadesmis confervacea Kützing 1844
Diatoma vulgaris Bory 1824
Diatoma vulgaris var. *linearis* Grunow 1881
Didymosphenia geminata (Lyngbye) Mart.Schmidt 1899
Dinobryon cylindricum O.E.Imhof 1887
Dinobryon divergens O.E.Imhof 1887
Diploneis puella (Schumann) Cleve 1894

Species name
Discostella asterocostata (B.Q.Lin, S.Q.Xie & S.X.Cai) Houk & Klee 2004
Dorofeyukea grimmei (Krasske) Kulikovskiy & Kociolek 2019
Encyonopsis lanceola (Grunow) Krammer 1997
Euglena tristella S.P.Chu 1946
Fallacia lenzii Lange-Bertalot 2004
Fragilaria amphicephaloides Lange-Bertalot 2013
Pseudostaurosira brevistriata var. elliptica (Héribaud) J.C.Kingston 2000
Frustulia rhomboides (Ehrenberg) De Toni 1891
Geissleria acceptata (Hustedt) Lange-Bertalot & Metzeltin 1996
Geitlerinema splendidum (Gomont) Anagnostidis 1989
Gloeotila subconstricta (G.S.West) Printz 1964
Gogorevia exilis (Kützing) Kulikovskiy & Kociolek 2020
Gomphonella calcarea (Cleve) R.Jahn & N.Abarca 2019
Gomphonella olivacea (Hornemann) Rabenhorst 1853
Gomphonema acuminatum Ehrenberg 1832
Gomphonema constrictum Ehrenberg 1844
Gomphonema sphaerophorum Ehrenberg 1845
Grunowia tabellaria (Grunow) Rabenhorst 1864
Gyrosigma acuminatum (Kützing) Rabenhorst 1853
Gyrosigma attenuatum (Kützing) Rabenhorst 1853
Halamphora dusenii (Brun) Levkov 2009
Hannaea arcus (Ehrenberg) R.M.Patrick 1966
Hantzschia amphioxys (Ehrenberg) Grunow 1880
Hippodonta capitata (Ehrenberg) Lange-Bertalot, Metzeltin & Witkowski 1996
Hyaloraphidium rectum Korshikov 1953
Iconella biseriata (Brébisson) Ruck & Nakov 2016
Kamptonema formosum (Bory ex Gomont) Strunecký, Komárek & J.Smarda 2014
Klebsormidium subtile (Kützing) Mikhailyuk, Glaser, Holzinger & Karsten 2015
Kurtkrammeria aequalis (W.Smith) Bahls 2015
Leibleinia gracilis (Rabenhorst ex Gomont) Anagnostidis & Komárek 1988
Leptolyngbya lagerheimii (Gomont) Anagnostidis & Komárek 1988
Limnospira maxima (Setchell & N.L.Gardner) Nowicka-Krawczyk, Mühlsteinová & Hauer 2019
Lindavia bodanica (Eulenstein ex Grunow) T.Nakov, Guillory, Julius, Theriot & Alverson 2015
Lobochlamys segnis (H.Ettl) Pröschold, B.Marin, U.W.Schlösser & Melkonian 2001
Luticola goeppertiana (Bleisch) D.G.Mann ex Rarick, S.Wu, S.S.Lee & Edlund 2017
Mayamaea atomus (Kützing) Lange-Bertalot 1997
Melosira varians C.Agardh 1827
Meridion circulare (Greville) C.Agardh 1831
Merismopedia glauca (Ehrenberg) Kützing 1845
Merismopedia sinica S.-H.Ley 1947
Merismopedia tranquilla (Ehrenberg) Trevisan 1845

Species name
Microcoleus amoenus (Gomont) Strunecky, Komárek & J.R.Johansen 2013
Microspora irregularis (West & G.S.West) Wichmann 1937
Monactinus simplex (Meyen) Corda 1839
Monoraphidium contortum (Thuret) Komárková-Legnerová 1969
Monoraphidium minutum (Nägeli) Komárková-Legnerová 1969
Monoraphidium mirabile (West & G.S.West) Pankow 1976
Mougeotia scalaris Hassall 1842
Myriactis pulvinata Kützing 1843
Navicula adversa Krasske 1938
Navicula angusta Grunow 1860
Navicula phyllepta Kützing 1844
Navicymbula pusilla (Grunow) Krammer 2003
Neidiomorpha binodis (Ehrenberg) M.Cantonati, Lange-Bertalot & N.Angeli 2010
Neidium kozlowii Mereschkowsky 1906
Nitzschia acicularis (Kützing) W.Smith 1853
Nitzschia palea (Kützing) W.Smith 1856
Nodularia spumigena Mertens ex Bornet & Flahault 1888
Nostoc verrucosum Vaucher ex Bornet & Flahault 1886
Odontidium hyemale (Roth) Kützing 1844
Odontidium mesodon (Ehrenberg) Kützing 1849
Oscillatoria princeps Vaucher ex Gomont 1892
Oscillatoria tenuis C.Agardh ex Gomont 1892
Pandorina morum (O.F.Müller) Bory 1826
Pantocsekiella ocellata (Pantocsek) K.T.Kiss & Ács 2016
Pectinodesmus javanensis (Chodat) E.Hegewald, C.Bock & Krienitz 2013
Phormidium allorgei (Frémy) Anagnostidis & Komárek 1988
Phormidium papyraceum Gomont 1892
Phormidium willei (N.L.Gardner) Anagnostidis & Komárek 1988
Pinnularia bihastata (A.Mann) F.W.Mills 1934
Placoneis dicephala (Ehrenberg) Mereschkowsky 1903
Planktolyngbya limnetica (Lemmermann) Komárková-Legnerová & Cronberg 1992
Planktothrix agardhii (Gomont) Anagnostidis & Komárek 1988
Planothidium heidenii (P.Schultz) Witkowski, Lange-Bertalot & Metzeltin 2000
Pseudopediastrum boryanum (Turpin) Hegewald 2005
Pseudostaurosira brevistriata (Grunow) D.M.Williams & Round 1988
Scenedesmus quadricauda (Turpin) Brébisson 1835
Schroederia nitzschioides (G.S.West) Korschikov 1953
Sellaphora rectangularis (W.Gregory) Lange-Bertalot & Metzeltin 1996
Sellaphora seminulum (Grunow) D.G.Mann 1989
Sphaeroplea annulina (Roth) C.Agardh 1824
Spirogyra crassispina C.-C.Jao 1939
Spirogyra varians (Hassall) Kützing 1849
Spirulina major Kützing ex Gomont 1892

Species name
Staurastrum indentatum West & G.S.West 1902
Stauridium tetras (Ehrenberg) E.Hegewald 2005
Stauroneis javanica (Grunow) Cleve 1894
Staurosira inflata (Heiden) A.Rusanov, Ács, E.Morales & Ector 2018
Staurosirella lapponica (Grunow) D.M.Williams & Round 1987
Stephanocyclus meneghinianus (Kützing) Kulikovskiy, Genkal & Kociolek 2022
Stigeoclonium tenue (C.Agardh) Kützing 1843
Surirella angusta Kützing 1844
Surirella librile (Ehrenberg) Ehrenberg 1845
Surirella linearis var. *linearis* W.Smith 1853
Synechococcus ambiguus Skuja 1937
Synedra dorsiventralis O.Müller 1910
Synedra tabulata var. *rostrata* (Juhlin-Dannfelt) A.Cleve 1953
Ulnaria ulna (Nitzsch) Compère 2001
Tabularia affinis (Kützing) Snoeijs 1992
Tabularia tabulata (C.Agardh) Snoeijs 1992
Tetradesmus dimorphus (Turpin) M.J.Wynne 2016
Tychonema bornetii (Zukal) Anagnostidis & Komárek 1988
Tryblionella littoralis (Grunow) D.G.Mann 1990
Ulnaria acus (Kützing) Aboal 2003
Ulnaria contracta (Østrup) E.A.Morales & M.L.Vis 2007
Ulothrix tenuissima Kützing 1833
Ulothrix zonata (F.Weber & Mohr) Kützing 1833
Uronema confervicola Lagerheim 1887
